# Phthiocerol Dimycocerosates of *M. tuberculosis* Participate in Macrophage Invasion by Inducing Changes in the Organization of Plasma Membrane Lipids

**DOI:** 10.1371/journal.ppat.1000289

**Published:** 2009-02-06

**Authors:** Catherine Astarie-Dequeker, Laurent Le Guyader, Wladimir Malaga, Fam-Ky Seaphanh, Christian Chalut, André Lopez, Christophe Guilhot

**Affiliations:** 1 CNRS, Institut de Pharmacologie et de Biologie Structurale (IPBS), Toulouse, France; 2 Université de Toulouse, UPS, IPBS, Toulouse, France; University of Pittsburgh School of Medicine, United States of America

## Abstract

Phthiocerol dimycocerosates (DIM) are major virulence factors of *Mycobacterium tuberculosis* (*Mtb*), in particular during the early step of infection when bacilli encounter their host macrophages. However, their cellular and molecular mechanisms of action remain unknown. Using *Mtb* mutants deleted for genes involved in DIM biosynthesis, we demonstrated that DIM participate both in the receptor-dependent phagocytosis of *Mtb* and the prevention of phagosomal acidification. The effects of DIM required a state of the membrane fluidity as demonstrated by experiments conducted with cholesterol-depleting drugs that abolished the differences in phagocytosis efficiency and phagosome acidification observed between wild-type and mutant strains. The insertion of a new cholesterol-pyrene probe in living cells demonstrated that the polarity of the membrane hydrophobic core changed upon contact with *Mtb* whereas the lateral diffusion of cholesterol was unaffected. This effect was dependent on DIM and was consistent with the effect observed following DIM insertion in model membrane. Therefore, we propose that DIM control the invasion of macrophages by *Mtb* by targeting lipid organisation in the host membrane, thereby modifying its biophysical properties. The DIM-induced changes in lipid ordering favour the efficiency of receptor-mediated phagocytosis of *Mtb* and contribute to the control of phagosomal pH driving bacilli in a protective niche.

## Author Summary

*Mycobacterium tuberculosis (Mtb)*, the causative agent of human tuberculosis, is an extremely successful human pathogen. The pathogenesis of bacterium is associated with its ability to invade macrophages and to circumvent bactericidal functions of the host cell in order to survive within a protective niche. The cellular mechanisms are largely investigated but the bacterial factors are poorly known. The outermost layer of the mycobacterial cell envelope is particularly of interest because of its localization at the interface with macrophages. An interesting feature of this envelope is its high lipid content. One group of lipids, the phthiocerol dimycocerosates (DIM), has been studied intensively since being shown to promote *Mtb* virulence. We investigated the cellular and molecular mechanisms of DIM and demonstrated that DIM participate in the receptor-dependent phagocytosis of *Mtb* in human macrophages through a mechanism involving a reorganization of the plasma membrane following recognition of bacilli. This modification of the plasma membrane biophysical properties might help *Mtb* to create a protective niche by preventing acidification of its phagosome. Our results provide a first hint on the molecular mechanism of action of DIM, a key *Mtb* lipidic virulence factor.

## Introduction

The cell envelope of *Mycobacterium tuberculosis* (*Mtb*) plays a major role in the pathogenesis of this bacterium [1]. An interesting feature of this envelope is its high lipid content, constituting up to 40% of the dry weight of mycobacteria [2]. One group of lipids, phthiocerol dimycocerosates (DIM), has been studied intensively since being shown to promote *Mtb* virulence. DIM are produced by all members of the *Mtb* complex and a few other mycobacterial species, most pathogenic in humans or animals. They are produced by the combined action of fatty acid synthases and polyketide synthases and in *Mtb* are composed of a mixture of long-chain β-diols esterified by multimethyl-branched fatty acids named mycocerosic acids [3]. They are found near the surface of the bacilli [4] and are thought to be part of the mycobacterial pseudo-outer membrane [1],[5]. They play a role in cell wall permeability [6] and many studies have implicated these molecules in the pathogenicity of *Mtb*. Indeed, A DIM-less *Mtb* strain was initially shown to display lower levels of replication and to elicit fewer lung surface tubercles than a DIM-producing strain [7]. An avirulent strain of *Mtb* coated with a mixture of DIM and cholesteryl oleate persisted longer than the uncoated strain in the spleen and lung of infected mice [8]. Consistent with these initial observations, two independent signature-tagged transposon mutagenesis studies have led to the isolation of *Mtb* mutants with a severe growth defect in mice [9],[10]. In some of these mutants, a transposon was found to have inserted into a region of the genome dedicated to the synthesis and transport of DIM, leading to a defect in DIM production or translocation. These studies clearly demonstrated the importance of DIM production for *Mtb* pathogenicity. However, it remains unclear whether DIM mediate pathogenesis indirectly by changing the cell wall envelope or whether they act as effectors modifying host immune responses and notably interfering with antimicrobial activity.

Recent studies have suggested that DIM play a role in modulating the early immune responses of murine macrophages. Rousseau et al. showed that DIM production in *Mtb* contributes to the initial growth of the bacillus in mice by protecting it from the nitric oxide-dependent killing of macrophages and modulates the production of key inflammatory cytokines such as TNFα [11]. A screening for mycobacterial mutants with defects in phagosomal acidification or maturation arrest in macrophages identified multiples genes including some involved in DIM synthesis [12],[13]. These studies, although small in number, suggest that DIM are involved in modulating the immune responses of murine macrophages, although the cellular and molecular mechanisms by which DIM participate in the cross-talk between *Mtb* and macrophages remain unclear.

The delivery of *Mtb*, and of particles in general, to the phagosome and the initiation of bactericidal responses requires an encounter with macrophages and ingestion through a receptor-mediated, actin-dependent process called phagocytosis. Mycobacteria make use of the phagocytic machinery to gain access to the interior of the macrophage where they subvert phagosome maturation. At the interface between mycobacteria and macrophages, the complex outermost layer of the mycobacterial cell wall probably plays a role in facilitating host cell entry. Some envelope molecules tested in competition assays with bacteria or coated on latex beads have been shown to interact with macrophage phagocytic receptors. For instance, the mycobacterial lipoarabinomannan and phosphatidylinositol mannosides bind to the mannose receptor (MR) [14],[15], one of the major receptors for mycobacterial entry, via the glycosylated part of the molecule [16],[17]. Another important phagocytic receptor of macrophages, the complement receptor 3, CR3 [18], recognizes outer mycobacterial capsular polysaccharides through its lectin site [19] and phosphatidylinositol mannosides [20].

DIM are located in the outer layer of *Mtb* cell envelope and therefore constitute potential candidates for modulating the initial step of entry into macrophages and for controlling the outcome of bacterial infection. We investigate the mechanism of action of DIM during host cell infection. We demonstrated the involvement of DIM in the receptor-dependent phagocytosis of *Mtb* through a mechanism involving plasma membrane reorganisation after bacteria recognition. This modification might enable *Mtb* to create a protective niche, by preventing phagosome acidification.

## Results

### DIM deficiency affects macrophage infection

**Figure 1:**
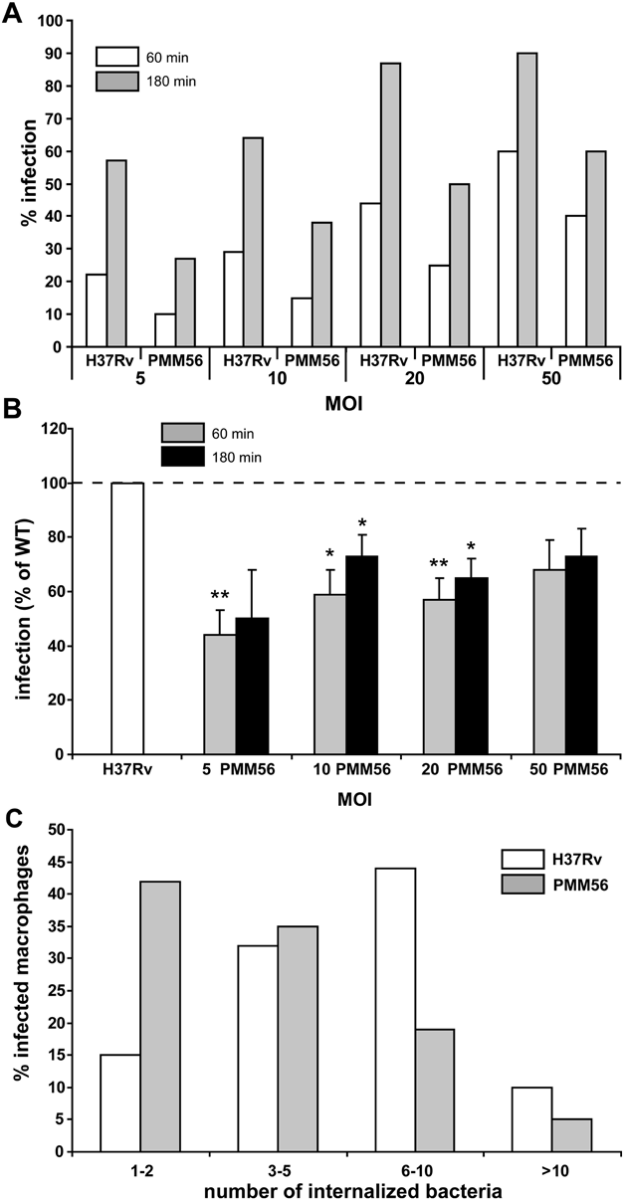
DIM are involved in the infection of human macrophages by *Mtb:* MDMs were incubated with H37Rv WT or a DIM-less PMM56 mutant and processed for counting by fluorescence microscopy. (A) Percentage of macrophages having ingested at least one bacterium after 60 or 180 minutes of incubation. Data from one experiment performed in duplicate and representative of three independent experiments. (B) Percentage of macrophages infected by the PMM56 mutant expressed with respect to the percentage of macrophages infected with the WT (H37Rv, set at 100%). The values are means±SEM of three to five separate experiments performed in duplicate. The significance of differences between the mutant and the WT strain was evaluated: *, p<0.05; **, p<0.02. (C) Percentage of infected macrophages after 180 minutes of incubation as a function of the number of ingested WT or the PMM56 mutant. Data from one experiment performed in duplicate and representative of two independent experiments.

We investigated the role of DIM in the infection of macrophages derived from monocytes (MDMs) by *Mtb* in a bacterial context by comparing the capacity of cells to take up the parental strain H37Rv (WT) and the DIM-deficient H37Rv mutant in which the *ppsE* gene has been deleted (PMM56, Table 1). Culture MDMs were used to mimic the initial encounter of bacilli with their host cells. The WT and mutant strains were transformed with pMV361H *gfp* which carries a hygromycin resistance cassette as the selection marker and a *gfp* gene for fluorescence microscopy analysis. As shown in Figure 1A and 1B, the DIM-less mutant infected fewer MDMs than did the WT, whatever the multiplicity of infection (MOI 5 to 50) and the duration of infection (60 or 180 minutes). After 1 h of infection, the PMM56 mutant had infected about half as many macrophages as H37Rv (Figure 1B). Analysis of the intracellular distribution of bacteria indicated that most infected macrophages contained 6 to 10 H37Rv per cell, but only 1 to 2 PMM56 mutants (Figure 1C). These preliminary observations suggest that DIM deficiency impaired the capacity of *Mtb* to infect MDMs. The effect of this deletion decreased slightly with increasing MOI or time of infection. We therefore carried out all the subsequent experiments at an MOI of 10 or 20 using an incubation time of 1 hour.

**Table 1:** Name and main features of strains, plasmids, and primers used in this study.

	Name	Relevant Characteristics	Reference/Source
**Strains**	H37Rv	*M. tuberculosis*	[67]
	BCG	*M. bovis* BCG 1173 P2 (Pasteur strain)	
	PMM56	*M. tuberculosis* H37Rv Δ*ppsE::res-Ωkm-res*	[48]
	PMM100	*M. tuberculosis* H37Rv Δ*ppsE::res*	This study
	PMM3	BCG Δ*pks15/1::res-Ωkm-res*	[47]
	PMM50	BCG Pasteur Δ*ppsE::res-Ωkm-res*	This study
	PMM97	BCG Pasteur Δ*ppsB::res*	This study
**Plasmids, phages**	pYUB854	Cosmid vector containing λ *cos* site	[63]
	phAE87	Shuttle phasmid derived from mycobacteriophage TM4	[63]
	phWM01	Shutle phasmid derived from phAE87 and containing *ΔppsE::res-Ωkm-res*	This study
	phWM07	Shutle phasmid derived from phAE87 and containing *ΔppsB::res-Ωkm-res*	This study
	pWM19	Thermosensitive *E. coli*/mycobacteria shuttle plasmid containing the resolvase gene from transposon γδ	[64]
	pMV361H	Integrative *E. coli*/mycobacteria shuttle vector	[65]
	pMIP12	Replicative *E. coli*/mycobacteria shuttle plasmid	[66]
	pMVE	Mycobacterial plasmid derived from pMV361H containing the *ppsE* gene	This study
	pMV361H *gfp*	Mycobacterial plasmid derived from pMV361H containing the *gfp* gene	D. Zerbib
	pCG211	Mycobacterial plasmid derived from pMIP12 containing the *gfp* gene	This study
**Primers**	ppsE-ATG	5′- ACATATGACGGGAAGCATCAGTGG-3′	
	ppsE-Stop	5′-AACAAAGCTTAGCCGGCATCCATGCTCGAC-3′	
	ppsB1	5′-TTGTTTAAACTGGTGACGAGCATGACCGG-3′	
	ppsB2	5′-TTGATATCACCCTGTCGAACTCGTCGG -3′	
	ppsB5	5′-ATATGATATCGGAATCTATCTGGCGGCCTTTG -3′	
	ppsB6	5′-TATATCTAGAGTTTAAACTCCGAAAGTTGTTCCAACAACTCG -3′	
	ppsB7	5′-GCGATCAACGACGACTTAGG -3′	
	ppsB8	5′-GGCATGTTCGAGTGCTTCC -3′	
	ppsB9	5′-CGCATCTCATCCGACAGCAGCT -3′	
	ppsB10	5′-GGTGTGATCATCACAGTAGTAG -3′	
	gfp1	5′-ATGGGATCCACAATTGTGAGCAAGGGCGAG-3′	
	gfp2	5′-TTACTAAGTACGCTAGTTAACTACGTCGAC-3′	

We investigated the poorly infectious phenotype associated with DIM deficiency, and sought to confirm this phenotype using various recombinant strains. An unmarked DIM-less mutant derived from PMM56 was constructed by excision of the *res-Ωkm-res* cassette (Table 1). Like the parental strain PMM56, PMM100 was unable to synthesize DIM (Figure S1). The *ppsE* mutation was then complemented by the transfer of pMVE allowing expression of the *ppsE* gene (Table 1). As expected, this plasmid restored DIM production (Figure S1). The capacity of the unmarked mutant and the complemented strain to infect MDMs was then compared with that of the WT strain and of the PMM56 mutant. As expected, the marked and unmarked DIM-less mutants were similarly affected in their capacity to infect MDMs whereas the genetic complementation of the *ppsE* mutation almost completely rescued the WT phenotype (Table 2), indicating that the response was essentially due to the gene deletion. We also constructed a mutant strain derived from *M. bovis* BCG with the same *ppsE* mutation as the PMM56 mutant (Table 1). We then analyzed the capacity of this new *M. bovis* BCG mutant, named PMM50, to infect MDMs. We found that the deletion of *ppsE* in *M. bovis BCG* abolished the synthesis of DIM (Figure S1) and decreased the efficiency with which bacteria infected MDMs (Table 2). The *ppsE* deletion also decreased the rate of infection of PMA-differenciated THP-1 by *M. bovis* BCG (Table 2).

**Table 2:** *ppsE* mutation decreases the ability of *M. tuberculosis* complex strains to infect human macrophages.

	MDMs		THP-1	
	Infected Macrophages (%)	Infection (% WT)	Infected Macrophages(%)	Infection (% WT)
BCG	20±3 (n = 4)	100	46±5 (n = 2)	100
PMM50	12±4 (n = 4)	60±4***	22+4 (n = 2)	48±4
H37Rv	66±6 (n = 3)	100		
PMM56	29±8 (n = 3)	44±9 *		
PMM100	31±5 (n = 3)	47±6**		
PMM100pMVE	56±10 (n = 3)	85±9 ^+^		

MDMs or PMA-differentiated THP-1 were incubated for 60 minutes with strains at an MOI of 10. The significance of differences between the mutant and the WT strain was evaluated: *, p<0.05; **, p<0.01; ***, p<0.001, or between *ppsE*-complemented strain and the PMM100 mutant: +, p<0.05.

Thus the *ppsE* mutation and the resulting DIM deficiency, decrease the ability of *M. tuberculosis* complex strains to infect macrophages.

### DIM directly contribute to the initial step of macrophage infection

**Figure 2:**
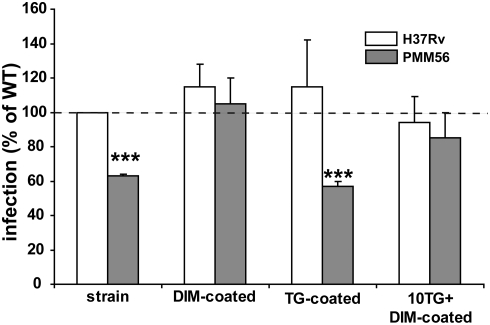
DIM contribute directly to macrophage infection by *Mtb.* MDMs were incubated for 60 minutes with strains at an MOI of 10: solvent-treated H37Rv WT or the PMM56 mutant uncoated or coated with DIM or triglycerides (TG) or DIM with excess TG. Data are expressed as a percentage infection with respect to the WT (H37Rv; 100%). The values are means±SEM of three separate experiments. We evaluated the significance of difference between the WT strain and the mutant or TG-coated mutant: *** p<0.01.

As DIM were thought to be involved in mycobacterial cell envelope organization, it was therefore hypothesized that their loss might alter the surface properties of *Mtb*, thereby affecting the macrophage invasion process. We investigated this issue by purifying DIM from H37Rv and using them to coat the PMM56 mutant or the WT strain. We then compared the invasion efficiency of the coated and uncoated strains. Coating with DIM had no significant effect on H37Rv uptake but allowed the PMM56 mutant to infect macrophages as efficiently as the parental strain (Figure 2).

We investigated whether this effect on phagocytosis was specific to DIM or applied to all apolar lipids, by carrying out the same experiment with triglycerides (TG). The coating of the PMM56 mutant with TG had no significant effect on bacterial internalization (Figure 2). When used in excess, to compete with DIM for coating of the PMM56 mutant, TG did not significantly prevent the reversion of the mutant phenotype induced by DIM alone (Figure 2). Thus, DIM seem to have a direct effect on the host cell rather than simply changing/altering bacterial cell wall organization. In line of this hypothesis, the *M. tuberculosis* mutant, displaying changes in envelope organization due to a mutation within the mycoloyltransferase gene *fbpC* [21], was internalized to a similar extent to the parental WT strain (data not shown). The data provided evidence for a role of DIM in the initial step of macrophage infection by *Mtb*.

### DIM participate in the receptor-dependent phagocytosis of *Mtb*

**Figure 3:**
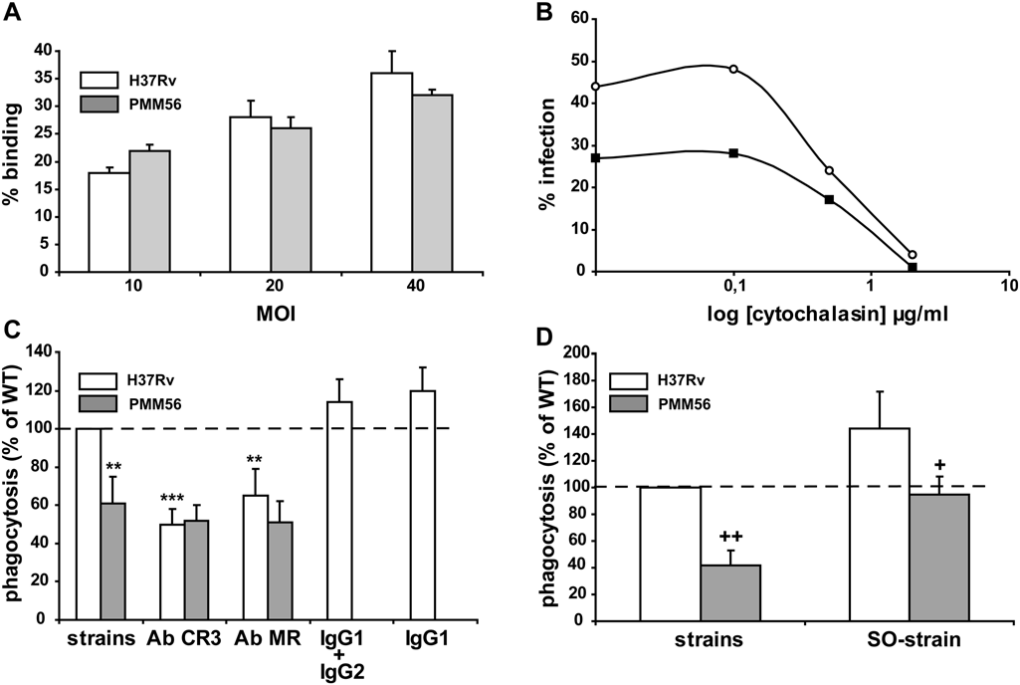
DIM participate in the control of the receptor-dependent phagocytosis of H37Rv. (A) MDMs were incubated for 30 minutes at 4°C with the WT or the PMM56 mutant, at various MOI, and binding was determined by assessing immunofluorescence. Data are presented as the percentage of macrophages having bound at least one bacterium; one experiment that is representative of two independent experiments. (B) MDMs were incubated at 37°C for 30 minutes with various concentrations of cytochalasin and were then infected with either H37Rv WT (white circle) or the PMM56 mutant (black square) at an MOI of 10. Data are presented as percentage phagocytosis; one experiment representative of two independent experiments. (C) MDMs were incubated at 37°C for 30 minutes with irrelevant IgG1 or a cocktail of IgG1 and IgG2 or with mAbs directed against CR3 or MR. They were then washed and infected for 60 minutes with H37Rv WT or the PMM56 mutant at an MOI of 10. Data are presented as the percentage of phagocytosis compared to WT value (100% H37Rv). (D) MDMs were infected for 60 minutes with serum-opsonized (SO-strain) or non opsonized strains H37Rv WT or the PMM56 mutant at an MOI of 10. Data are presented as the percentage phagocytosis with respect to WT (H37Rv; 100%). The values presented are means±SEM of three independent experiments. The significance of difference between mAb-treated cell and untreated cells was evaluated: *, p<0.05; **, p<0.02. We also evaluated the significance of difference between mutants and the WT strain: +, p<0.05; ++, p<0.01.

It is generally accepted that mycobacteria are taken up into macrophages through a conventional phagocytic process [22] that can be divided into two steps: i) the recognition and the adhesion through binding to surface receptors and ii) the integration of signaling pathways triggering local remodeling of the actin cystoskeleton and leading to particle engulfment. We investigated the possible role of DIM in the adhesion and/or the uptake of *Mtb*, by placing MDMs in contact with H37Rv or the PMM56 mutant and incubating for 30 minutes at 4°C to allow particles to bind, whilst preventing phagocytosis. At the end of the incubation period, non-adherent extracellular bacteria were eliminated by washing the cells with fresh cold medium and bound bacilli were detected by incubation with an antibody directed against mycobacteria. We counted the cells that had bound bacilli and found that the WT and PMM56 strains adhered to macrophages to a similar extent (19±6 and 23±7% binding, n = 3 at MOI 10) regardless of the MOI used (Figure 3A).

We found that the uptake of both WT and PMM56 strains required an intact actin filament network. Indeed, the prior treatment of macrophages with cytochalasin, an actin-depolymerizing drug, inhibited the internalization of the WT and the PMM56 strains in a dose-dependent manner, with a maximal effect at 2 µg/ml (80±7% and 77±12% inhibition, n = 3, respectively) (Figure 3B).

Actin is also required for macropinocytosis, a mechanism by which mycobacteria have recently been shown to invade macrophages [23] or non phagocytic cells, such as type II (A549) human pneumocytes [24]. Following the infection of A549 cells for 2 hours at an MOI of 20 with the WT strain or the PMM56 mutant, we found that both strains infected a similar percentage of cells (51±6% and 49±7%, (n = 3), respectively). Thus, the lower capacity for the mutant uptake is probably restricted to macrophages (MDMs and PMA-differentiated THP-1) strongly suggesting that DIM affect the receptor-dependent phagocytosis of *Mtb* rather than macropinocytosis in human macrophages.

We then investigated whether the absence of DIM specifically affected the entry of H37Rv into human macrophages through a particular receptor. The major receptors used by *Mtb* for the invasion of MDMs are the complement receptor CR3 (CR3) and the mannose receptor (MR) [14],[18]. We assessed the uptake of the WT strain or of the PMM56 mutant using blocking antibodies raised against human CR3 or MR. We used a combination of two blocking anti-CR3 Abs ICRF 44 and D12 [25],[26] or the CD206 anti-MR mAb [27] or irrelevant mouse anti-human immunoglobulins IgG1 and IgG2 as isotype controls. We tested various dilutions of mAbs and efficiency was highest for 100 and 50 µg/ml for ICRF44 and D12, respectively, with a 50±8% decrease in H37Rv internalization (Figure 3C), and for 2 µg/ml for anti-MR mAb resulting in a 35±14% decrease in H37Rv uptake (Figure 3C). By contrast to the phagocytosis of the WT strain, no inhibition of the PMM56 mutant was observed with any of the blocking antibodies (Figure 3C). These results were extended to phagocytosis via the opsonic receptor. Serum opsonization of the WT strain or the PMM56 mutant increased the capacity of these strains to infect macrophages, but phagocytosis of the opsonized mutant remained less efficient than that of the opsonized WT (Figure 3D), indicating that DIM deficiency also impaired the activity of opsonic receptors.

These results demonstrate the need for DIM for the receptor-dependent phagocytosis of *Mtb* by MDMs, targeting the uptake rather than bacterial adhesion, and show that the effect of DIM was not restricted to a specific receptor.

### The role of DIM in *Mtb* phagocytosis required plasma membrane cholesterol

Pioneering studies by Gatfield and Pieters [28] and Peyron et al. [29] have proposed that mycobacteria enter phagocytic cells by a cholesterol-dependent mechanism. As DIM did not act on a specific receptor, we reasoned that DIM might work in concert with plasma membrane cholesterol.

**Figure 4:**
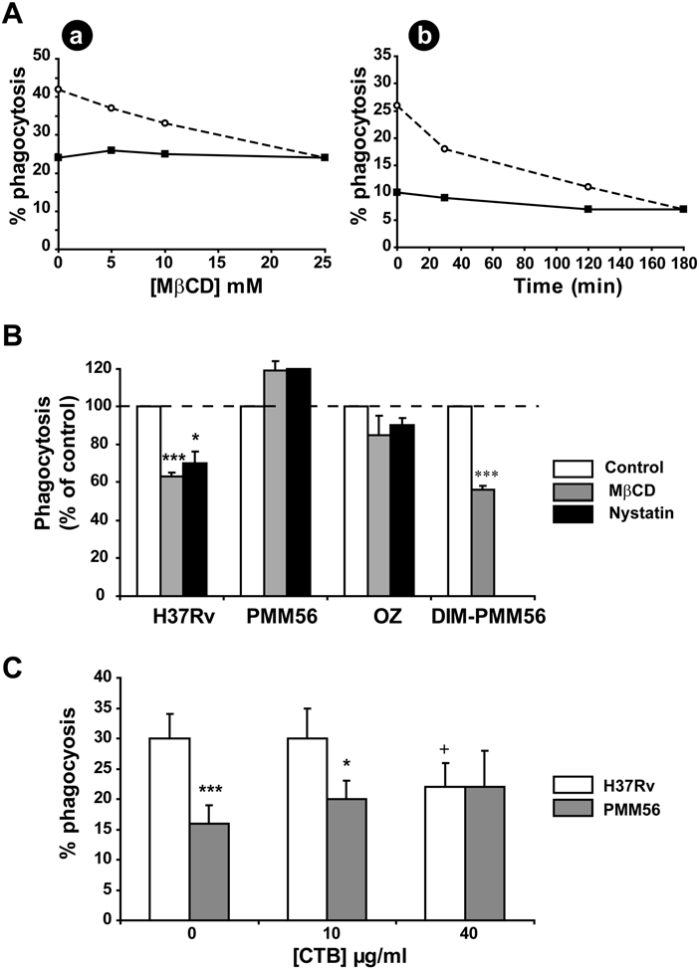
The DIM-dependent phagocytosis of *Mtb* required plasma membrane cholesterol. (A) MDMs were initially incubated at 37°C with (a) various concentrations of MβCD for 30 minutes or (b) 10 mM MβCD for various periods of time. They were then washed and infected for 60 minutes with H37Rv WT (white circle) or the PMM56 mutant at an MOI of 10 (black square); one experiment representative of three separate experiments. (B) MDMs left untreated or treated with 10 mM MβCD or 20 µg/ml Nystatin for 30 minutes were infected with H37Rv WT or the PMM56 mutant or the DIM-coated PMM56 mutant at an MOI of 10 for 60 minutes or incubated with the serum-opsonized zymosan (OZ) at an MOI of 25 for 45 minutes. The data are expressed as a percentage phagocytosis with respect to untreated conditions (control; 100%). The values presented are means±SEM for three to four separate experiments. We evaluated the significance between cell subjected to drug pretreatment and untreated cells: *, p<0.05; ***, p<0.01. (C) MDMs were left untreated or treated with various concentrations of CTB for 30 minutes. They were washed and infected for 60 minutes with H37Rv WT or the PMM56 mutant at an MOI of 10. The data are presented as the percentage of phagocytosis. The values are means±SEM for three separate experiments. The significance of the drug effect was assessed by comparing data under treated and untreated conditions: +, p<0.05; the significance of the effect of DIM deficiency was assessed by comparing data for mutants and the WT strain.*, p<0.05, ***p<0.01.

We tested this hypothesis by treating MDMs with a cholesterol-depleting molecule, methyl-β-cyclodextrin (MβCD). Following treatment with various concentrations of the drug for 30 minutes, the phagocytosis of H37Rv was found to be inhibited in a dose-dependent manner (Figure 4Aa). A similar inhibitory profile was obtained using a constant concentration of 10 mM MβCD but with an increasing incubation time (Figure 4Ab). By contrast, cholesterol depletion had no effect on internalization of the PMM56 mutant (Figure 4A and 4B). Similar results were obtained with another cholesterol-depleting drug, nystatin (Figure 4B). None of these drugs had any significant effect on serum-opsonized zymosan (Figure 4B), indicating the absence of a general effect on phagocytic functions in the experimental conditions used. Cholesterol depletion did not affect the binding of H37Rv or of the PMM56 mutant (32±11% and 37±21% or 32±11 and 31±8% binding, n = 2, in the absence and the presence of the drug, respectively). The involvement of DIM in H37Rv uptake was therefore dependent on cholesterol. Consistent with this, coating the PMM56 mutant with DIM restored the capacity of MβCD to inhibit bacterial entry (Figure 4B).

Pathogenic mycobacteria have recently been shown to promote the mobilization of cholesterol-enriched domains known as rafts, within macrophages, to the site of contact between the bacilli and the host cell [28],[30]. We investigated the role of raft lipids in H37Rv entry, by treating cells with a ganglioside GM1-binding molecule, cholera toxin subunit B (CTB) [31], which crosslinks GM1. The prior treatment of MDMs for 30 minutes with this drug decreased the phagocytosis of H37Rv in a dose-dependent manner, with a 28±3% decrease observed at a concentration of 40 µM (Figure 4C). By contrast, the level of internalization of the PMM56 mutant remained unchanged, or even slightly increased (Figure 4C). The entry of H37Rv into MDMs therefore required cholesterol and GM1 gangliosides whereas the uptake of the PMM56 mutant did not. This suggested that DIM might be involved in the use of lipid rafts by H37Rv to enter MDMs.

**Figure 5:**
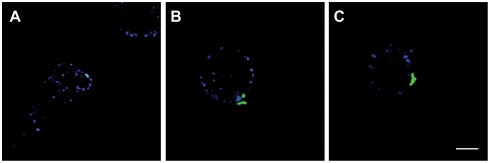
H37Rv associates weakly with plasma membrane domains enriched in GM1 gangliosides. H37Rv expressing GFP (green fluorescence) were allowed to adhere to MDMs for 30 minutes at 4°C. Cells were then stained with Alexa-647 CTB (blue fluorescence) and processed for analysis by confocal microscopy. Representative single optical *x-y* planes from confocal microscopy images are shown. (A) Colocalization of GFP-H37Rv and Alexa-647 CTB at the plasma membrane gives a turquoise signal. (B–C) Two examples of an absence of colocalization between GFP-H37Rv and Alexa-647 CTB at the plasma membrane. The data presented are from a single experiment representative of two. Bar: 12 µm.

We examined the distribution of GM1 gangliosides at the cell surface during the interaction with H37Rv, by placing MDMs in contact with bacilli and incubating at 4°C for 30 minutes, and then staining with Alexa-647 CTB for 1 hour at 4°C for 1 h. Confocal microscopy showed that GM1 gangliosides were present at the cell surface (Figure 5) and colocalized with adherent bacilli (Figure 5A), indicating that accumulation of this fluorescent marker could be detected. However, this colocalization was sporadic and most of the bacilli did not associate with GM1 ganglioside-enriched domains (Figure 5B and 5C), even when experiments were conducted at 37°C in conditions permissive for phagocytosis (data not shown). Similar data were obtained with filipin, a cholesterol-binding agent that displays blue fluorescence following its intercalation into the cell membrane (data not shown). Thus, if cholesterol or GM1 gangliosides are redistributed at WT entry foci, this phenomenon was either scarce in MDMs or was difficult to detect in our experimental conditions.

### DIM play a role in the lower acidity levels of *Mtb*-containing phagosomes

**Figure 6:**
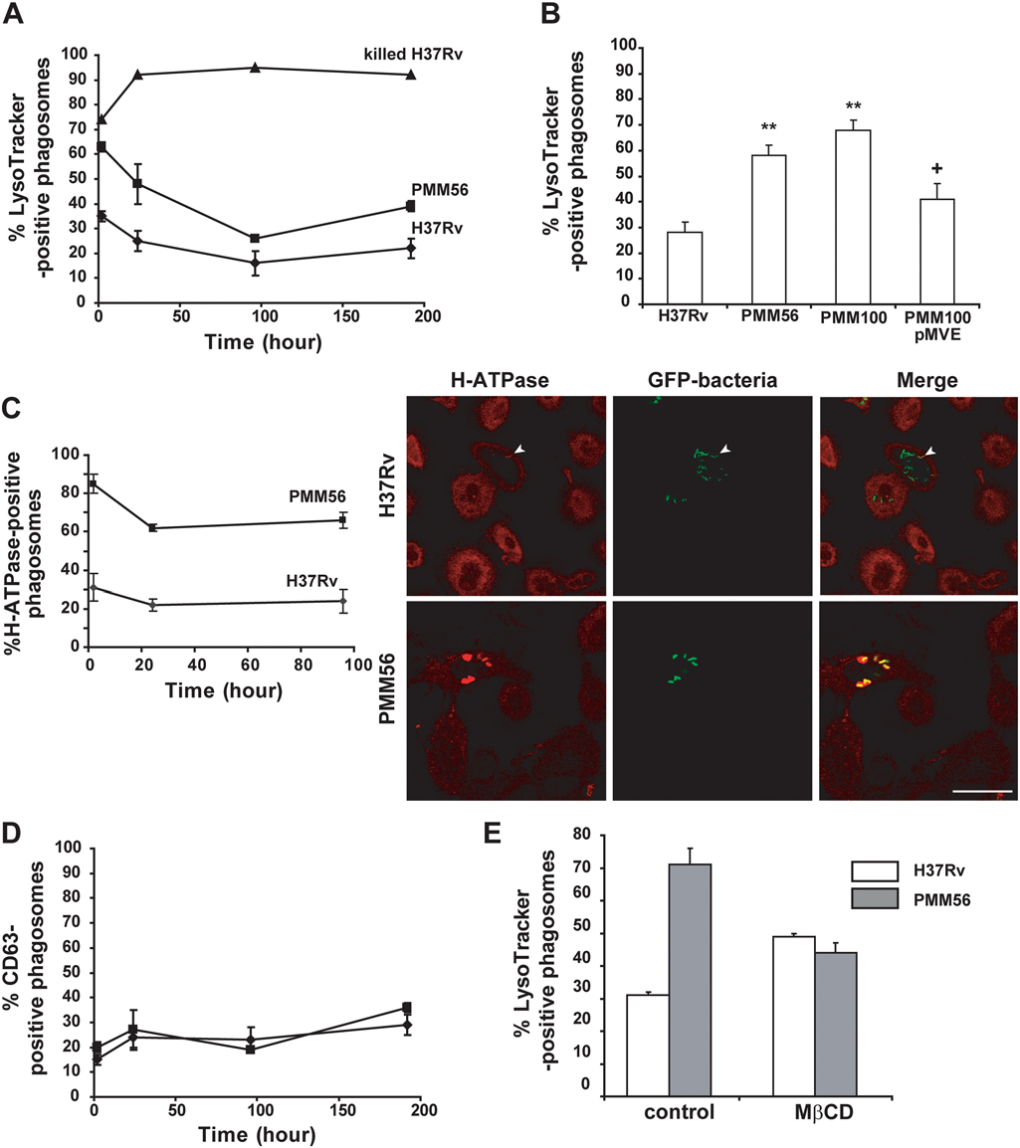
DIM deficiency induces acidification of *Mtb*-containing phagosome in a cholesterol-dependent manner. MDMs were infected for 60 minutes with heat-killed (triangles) or live H37Rv WT (diamonds) or the PMM56 mutant (square) at MOI 10, washed, and further incubated in the presence of serum. At various times after infection, MDMs were processed. (A,B,E) macrophages were added with LysoTracker, fixed, and processed for analysis by microscopy. LysoTracker-positive phagosomes were quantified over a period of 192 h (A) or 2 h after infection with H37Rv, PMM56, and PMM100 DIM-less mutants, or the complemented strain PMM100pMVE (B), or left untreated or treated with 10 mM MβCD before infection (E). (C–D) MDMs were fixed, permeabilized, and immunostained with (C) polyclonal anti-serum against H^+^-ATPase or (D) mAb against CD63 and processed for fluorescence microscopy and quantification. (C) A representative micrograph of cells analysed by confocal microscopy. Colocalization of H37Rv and H^+^-ATPase gave a yellow signal. Arrowheads indicated phagosome having occasionally accumulated H^+^-ATPase in MDMs infected by H37Rv. Bar: 34 µm. The percentage of phagosomes positive for each marker was determined by counting 100 phagosomes from at least ten different fields in duplicate samples. Data are means±SEM of two to four experiments. The significance of the effect was assessed by comparing data for mutants and the WT strain. **, p<0.01, or for the complemented strain and the unmarked mutant. +, p<0.05.

Our findings clearly indicated a role for DIM in the portal entry of *Mtb*. We then investigated the contribution of DIM to control the maturation of *Mtb*-containing phagosomes. It is generally thought that phagosomes containing live pathogenic mycobacteria are arrested at an early stage of maturation characterized by incomplete luminal acidification [32]. We characterized phagosomes containing H37Rv or the PMM56 mutant by investigating the colocalization of these strains with LysoTracker Red, an acidotropic dye acting as an indicator of phagosome pH [33]. MDMs were infected by incubation with H37Rv or the PMM56 mutant for 1 hour. They were then thoroughly washed with fresh medium and analyzed at different time points after infection. We found that 35% of H37Rv-containing phagosomes colocalized with LysoTracker at 2 hours (Figure 6A). This fraction decreased slightly to 25% at 24 h and remained constant thereafter over a period of 192 hours. By contrast, a much higher percentage of phagosomes containing the PMM56 mutant (63%, p = 0.01, n = 3) colocalized with LysoTracker soon after infection (2 h; Figure 6A). This percentage gradually decreased during phagosome maturation, but remained different from that of the WT over a period of 192 hours (p = 0.04, n = 3). In parallel experiments, we checked that LysoTracker colocalized with most of the phagosomes containing heat-killed H37Rv, used as control for normal phagosome-lysosome fusion (Figure 6A). Mean viability was 85% for both the WT and the mutant strains. This may account for the proportion of H37Rv accumulating the acidotropic dye but it cannot account for the difference between H37Rv and the PMM56 mutant. Genetic complementation of the *ppsE* mutation restored the WT phenotype (Figure 6B). Phagosomal acidification has been attributed principally to the activity of the vacuolar type H^+^-ATPase (V-ATPase), which has been shown to accumulate in the phagosomal membrane as it matures within the cell [34],[35]. Using an antibody directed against the H^+^-ATPase [36], we demonstrated that 20 to 30% of phagosomes containing H37Rv accumulated the H^+^-ATPase over a period of 96 hours (Figure 6C). In contrast, 80–90% of phagosomes containing PMM56 colocalized with the H^+^-ATPase soon after infection (Figure 6C). This percentage slightly decreased with the time of phagosome maturation but remained twice higher than the percentage of phagosomes containing the WT strain over 96 hours post-infection (Figure 6C). These data are consistent with the accumulation of LysoTracker in phagosome containing PMM56.

We then examined whether LysoTracker and H^+^-ATPase accumulation in phagosome containing DIM-less mutant was associated with a maturation of phagosome toward a fusion with lysosomes, by analyzing the translocation of the membrane-associated glycoprotein, CD63, used as a marker of lysosomal compartment in human macrophages [37]. The fraction of phagosomes containing H37Rv colocalizing with CD63 ranged from 15±3 to 29±4% over a period of 192 hours, and no difference was observed between the WT and the PMM56 mutant (Figure 6D). Thus, the *ppsE* mutation drove a significant percentage of H37Rv cells toward a compartment that transiently accumulated LysoTracker in the first few hours after infection without fusion with CD63 positive-lysosomes.

The number of bacteria per MDM were also quantified using direct counting of fluorescent bacteria as described previously [38]. As depicted in Figure S2, no significant difference was found in the growth curves of PMM56 and H37Rv within macrophages. These data showed that DIM participate in the control of acidification of phagosome containing H37Rv but DIM deficiency did not either drive bacilli toward a lysosomal compartement or affected the ability of *M.tuberculosis* to replicate in human macrophages.

We investigated this transient process, by studying the colocalization between LysoTracker and phagosomes containing bacteria in macrophages treated with MβCD. Cholesterol depletion increased the percentage of colocalization between LysoTracker and phagosomes containing H37Rv (Figure 6E), indicating that cholesterol is required for the control of luminal pH as recently reported [39]. By contrast, cholesterol depletion decreased the colocalization of LysoTracker with phagosomes containing the PMM56 mutant, to level similar to those for the WT strain (Figure 6E). These findings suggest, as for phagocytosis, that both DIM and cholesterol were important for the control of phagosomal pH.

### DIM decrease the polarity of the plasma membrane in human macrophages

The previous findings demonstrated that DIM at the surface of the *Mtb* envelope interfered with phagocytosis and blocked phagosomal acidification in a cholesterol-dependent manner. Cholesterol depleting drugs not only disrupt lipid rafts, they also affect membrane fluidity [40]. Furthermore, the extraction of membrane cholesterol destroys liquid cholesterol-phospholipid complexes leaving solid-like-high melting point phospholipid domains and inhibiting the lateral diffusion of membrane components [41]. This suggested that the dynamic organization of the plasma membrane might be involved in the DIM-dependent phagocytosis of H37Rv. We therefore examined the effect of DIM on some of the physical properties of the plasma membrane and the molecular organization of its lipids, using the fluorescence properties of Py-met-chol [42]. This new cholesterol-pyrene probe combines the spectroscopic properties of pyrene as a sensor of the polarity of the membrane hydrophobic core and the capacity to mimic the lateral distribution of cholesterol. In model or biological membranes, the fluorescence spectrum of the monomer species of pyrene consists of five emission bands where *I*_1_ is a function of the polarity of the probe's surroundings and *I*_3_ is largely proportional to probe concentration. The *I*_1_/*I*_3_ fluorescence ratio therefore reflects the polarity of the membrane hydrophobic core [43] which is mostly correlated with the number of water molecules passively crossing the membrane [44]. Thus, changes in *I*_1_/*I*_3_ ratio reveal changes in lipid organisation, with a decrease ratio corresponding to a decrease in the permeability of membrane to water [45]. Py-met-chol also diplays a characteristic wide fluorescence band corresponding to the excimer species (*I*_E_) and the fluorescence *I*_E_/*I*_3_ fluorescence ratio is directly related to the dynamic lateral distribution of the probe in the membrane lipid network [46].

**Figure 7:**
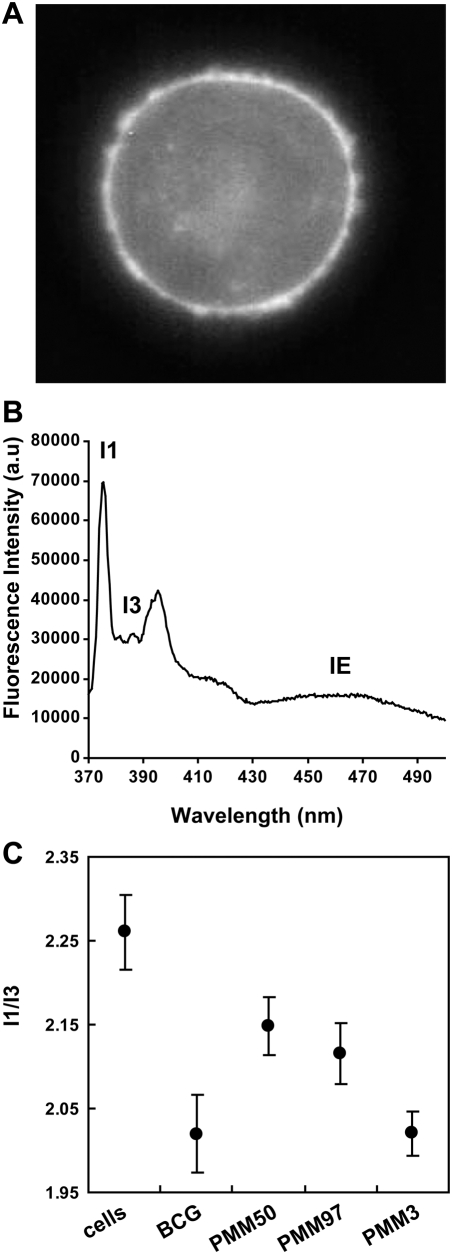
DIM contributes to the decrease in membrane polarity of THP-1 during the interaction with *M. bovis* BCG. (A) Py-met-chol was incorporated into the plasma membrane of THP-1, and cells were examined (A) by microscopy on a Zeiss Axioplan II using a DAPI Filter or (B) by spectrofluorimetry. (C) The *I*_1_/*I*_3_ fluorescence ratios were determined in THP-1 cells alone (n = 25) or after incubation of the cells for 30 minutes at room temperature with *M. bovis BCG* (n = 18), DIM-less mutants (PMM50, n = 15; PMM97, n = 11), or a PGL-less mutant (PMM3, n = 10), at an MOI of 10. Data are means±SD.

We first introduced Py-met-chol into the plasma membrane of intact living PMA-differentiated THP-1, using a probe-loaded MβCD, as previously described [42]. Under these conditions, no cholesterol depletion was observed [42]. Figure 7A and 7B showed a wide-field photomicrograph of probe-labelled cells and the corresponding fluorescence spectrum of a cell suspension, for which the *I*_E_/*I*_3_ ratio showed that about 1% of the Py-met-chol was inserted into the plasma membrane [42]. We then monitored the fluorescence spectra of Py-met-chol -loaded THP-1 brought into contact with *M. bovis* BCG or the isogenic PMM50 mutant for 30 minutes at room temperature, to limit bacterial phagocytosis. The interaction between THP-1 cells and *M. bovis* BCG resulted in a significantly lower *I*_1_/*I*_3_ ratio than obtained with THP-1 cells alone (Figure 7C). Thus, interactions between *M. bovis* BCG and THP-1 cells induce a decrease in global membrane polarity. Serum-opsonized zymosan used as a binding control also decreased *I*_1_/*I*_3_ ratio, but to a lesser extent than *M. bovis* BCG, despite similar numbers of bound particles per cell and a similar percentage of bound macrophages (data not shown). When the same experiment was performed with PMM50 mutant, the decrease in *I*_1_/*I*_3_ ratio was significantly smaller than that obtained with the WT strain (Figure 7C). This observation was not restricted to PMM50 mutant, because another isogenic strain with a mutation in the *ppsB* gene displayed a similar decrease in *I*_1_/*I*_3_ ratio (Figure 7C). By contrast, a mutant harbouring a *Δpks15/1::res-Ωkm-res* mutation, which has been shown to affect PGL synthesis without modifying DIM production [47], behaved like the WT strain. These findings demonstrate that DIM contributed to a decrease of the mean membrane polarity of THP-1 cells during interactions with *M.bovis* BCG. *I*_E_/*I*_3_ ratios did not differ between sets of conditions (data not shown), demonstrating an absence of dynamic reorganisation of Py-met-chol in the lipid phase of the plasma membrane during binding.

**Figure 8:**
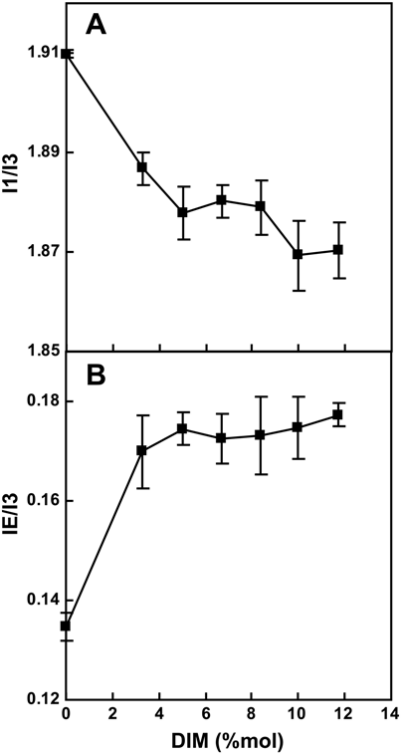
Insertion of DIM into model membranes decreases the polarity of the bilayer core by modifying membrane structure. DIM were incorporated into LUVs containing 1 mol% of Py-met-chol. Changes in the *I*_1_/*I*_3_ (A) and *I*_E_/*I*_3_ (B) fluorescence ratios of Py-met-chol in LUVs were examined as a function of DIM mole fraction. Measurements were performed in the fluid state, at 45°C. Data are means±SD of three independent experiments.

We got further insight into the mechanism by which DIM influence the polarity of the core of the plasma membrane, by incorporating DIM into model membranes (LUVs) containing 1 mol% of Py-met-chol. We chose DPPC as the main lipid as it gave optimal solubility of both DIM and probe, thanks to its fully saturated acyl-chains and length. We tested the incorporation of DIM into each sample, by carrying out dynamic light scattering measurements to detect small particles corresponding to aggregates. We found that the maximum solubility of DIM in DPPC was below 15 mol % (% mol/mol) (data not shown). We then carried out measurements of *I*_1_/*I*_3_ and *I*_E_/*I*_3_ (Figure 8) ratios of Py-met-chol as a function of DIM mole fraction (from 0 to 11.75 mol %) in fluid state LUVs. Once DIM were incorporated into LUVs, *I*_1_/*I*_3_ ratios decreased below those of LUVs without DIM and eventually reached a plateau as suggested by the error bars in Figure 8A. Thus, DIM insertion decreases the polarity of the bilayer core reflecting changes in the dynamic structure of the membrane. At the same time, *I*_E_/*I*_3_ ratio was considerably increased by the addition of small amount of DIM (Figure 8B). This addition resulted in confinement of the probe, inducing an increase in its apparent concentration, possibly due to the DIM-dependent recruitment of Py-met-chol into small domains. The increase in the amount of DIM from 5 to 11.75 mol % had no further effect on *I*_E_/*I*_3_ ratio (Figure 8B), indicating that DIM did not recruit /gather more cholesterol probes. This last observation seems to conflict with the data obtained in living cells. However, the plasma membrane has a mean cholesterol content of 30%, suggesting the dilution of Py-met-chol within the native cholesterol, making its lateral segregation more difficult to observe.

Taken together, these results showed that during the binding process, the presence of DIM at the mycobacterial cell surface induces the reorganisation of the host cell plasma membrane, probably through insertion into the membrane. This event led to a change in membrane properties and a more rigid (less polar and tighter) bilayer.

## Discussion

This work provides the first insight into a possible mechanism of action of DIM in *Mtb* pathogenesis. We demonstrated *ex-vivo* that DIM act in the initial cross-talk between human macrophages and *Mtb*. Our results strongly support a model in which contact of *Mtb* with phagocytes is followed by the insertion of DIM into the plasma membrane of the host, modifying the biophysical properties of this membrane. This modification increases the efficiency of receptor-mediated phagocytosis and affects phagosome acidification thereby facilitating the access of the pathogen to its ecological niche.

These conclusions were drawn from comparative studies of macrophage infection in a bacterial context using WT and mutant strains. These mutants harbour an insertion/deletion within the *ppsE* gene which encodes a protein essential for DIM biosynthesis [48]. The mutant phenotypes were attributed to the gene deletion and loss of DIM, because reversion to the WT phenotype was observed following genetic complementation of the mutation with an intact *ppsE* gene or chemical complementation with purified DIM. There are several lines of evidence to suggest that DIM play a direct role in the observed phenotypes rather than having an indirect effect through changes to the structural organization of the *Mtb* envelope. First, DIM are found at the mycobacterial cell surface, in an ideal location for direct interaction with the host cell [4]. Second, coating the PMM56 mutant with purified DIM restored the capacity of the bacteria to infect MDMs to WT levels. This phenotypic reversion would not have been observed if DIM acted indirectly by affecting mycobacterial membrane organization or the anchoring of other effector molecules. Third, we demonstrated that DIM inserted into model membranes *in vitro* modify the biophysical properties of this membrane in a similar manner to that observed with live bacteria and host cells.

Our results are consistent with a model in which the presence of DIM in the mycobacterial cell envelope directly interferes with host cell membrane functions. Other examples of the loss of cell surface molecules resulting in changes in the interaction between mycobacteria and host cells have been reported. For instance, the mechanical or enzymatic removal of capsular polysaccharides decreases *Mtb* binding to CHO cells expressing CR3 [19]. Disruption of the *Mtb* surface capsule by sonication significantly increases association with macrophages [49]. Diacyltrehaloses (DAT) and polyacyltrehaloses (PAT) deficiencies increase the efficiency of *Mtb* binding to phagocytic and non phagocytic cells [50]. This last observation was attributed to changes in the surface properties of DAT/PAT-deficient mutants including compositional changes and changes in net charges/hydrophobicity [50]. However, we reported herein that DIM deficiency did not affect binding to human macrophages. Instead, it interfered with a downstream event, receptor-mediated internalization. Indeed, prior treatment of the cells with blocking antibodies directed against CR3 or MR reduced WT strain uptake without affecting internalisation of the DIM-deficient mutant, indicating that the PMM56 mutant used another entry route. Similarly, prior incubation of PMM56 with serum did not induce a reversion of the rate of internalization to that observed with serum opsonized WT strain. We propose that this effect of DIM is mediated by direct modification of the host cell plasma membrane. Indeed, we demonstrated that changes in the composition of the MDMs plasma membrane induced by cholesterol depletion with MβCD or nystatin affected *Mtb* phagocytosis and phagosome acidification in a similar way to DIM elimination from the *Mtb* envelope. We also demonstrated the involvement of DIM in the decrease in plasma membrane core polarity following the binding of live mycobacteria. Consistent with these data for living cells, the incorporation of purified DIM into model membranes (LUVs) containing Py-met-chol decreased bilayer core polarity. The transfer of DIM from the *Mtb* cell envelope to the host cell membrane is also consistent with the finding of Beatty et al. [51] who showed that the mycoside B, a lipid from *M. bovis* BCG containing the same lipid core as DIM, was released from bacteria during the infection process and transported into various host cell organelles.

We characterized the DIM-dependent internalization of *Mtb* into macrophages by demonstrating that the more efficient uptake of WT strains than of DIM-less mutants seemed to be exclusively mediated by a receptor-dependent phagocytosis. Our data for experiments performed in the presence of cytochalasin indicated that both strains were internalized via an actin-dependent process. The actin-dependent mechanisms by which *Mtb* might be taken up into macrophages include macropinocytosis [24]. H37Rv was efficiently taken up by type II human pneumocytes, but the WT and the DIM-less mutants infected them to the same extent, suggesting that DIM enhance a receptor dependent phagocytic process in human macrophages. It remains unclear whether the entry process used influences the fate of the mycobacteria. We have previously demonstrated that the route of entry by which the mycobacteria invade macrophages favour their uptake in hospital conditions [52]. Consistent with this notion, we have observed that the absence of DIM in the envelope of H37Rv drove bacteria into phagosomes that accumulated an acidotropic dye early in infection. Our results are consistent with those of a previous study, in which a mutant lacking a DIM biosynthesis gene was unable to prevent vacuole acidification and acquired late endosomal markers, indicating that DIM-less mutants were defective in the arrest of phagosome maturation in murine macrophages [13]. The presence of DIM is required but not sufficient because we found that heat-killed bacteria which contained DIM accumulated in acidified compartment. These results imply that modulation of phagosome maturation is likely a multifactorial process that is mediated by other components in addition to DIM. Consistently, Stewart et al and Pethe et al identified multiple genes involved in mycobacterial control of early acidification of phagosomes [12],[13]. Further studies of the role of DIM in the arrest of phagosomes containing *Mtb* are required, because our data also indicate that both WT and mutant accumulated in a phagosome with low levels of expression of the lysosomal marker, CD63. Consistently DIM deficiency did not affect the ability of *M.tuberculosis* to replicate in human macrophages, as previously observed in murine macrophages [11]. It is therefore tempting to speculate that the presence of DIM at the surface of *Mtb* allows the preferential engagement of the portal of entry so as to participate in the early step of bacterial protection, possibly in collaboration with other components of *M.tuberculosis*.

Our results confirm that plasma membrane cholesterol is involved in bacterial entry and that plasma membrane cholesterol concentration is critical for the role of DIM in the phagocytosis of *Mtb*. Cholesterol is an integral constituent of lipid rafts which are thought to provide a entry portal for *Mtb* in murine macrophages [28]. For comparison, we studied the effect of the ganglioside GM1-binding molecule, CTB. The treatment of macrophages with this drug significantly decreased infection by H37Rv WT whereas no effect was observed on infection by a DIM-deficient mutant. However, investigation of the plasma membrane distribution of ganglioside GM1 or cholesterol by a fluorescence approach showed the occasional accumulation of a fluorescence signal at the site of impact of WT strains. Consistently, no Py-met-chol clustering was detected during the binding of bacteria to living cells. *Mtb* therefore seems to use lipid domains as rafts in human macrophages through a mechanism at least partly dependent on the presence of DIM at the surface of the bacillus but not involving the massive relocation and accumulation of gangliosides GM1 or cholesterol at the site of bacterial uptake, as previously reported in murine macrophages [28].

The mechanism by which DIM disturb plasma membrane and affect *Mtb* uptake remains unclear. DIM-dependent membrane alterations probably result from nonspecific lipid-lipid interaction rather than from the recognition of a specific molecular structure. As highlighted by Rhoades and Ullrich, the hydrophobic nature of mycobacterial lipids allows them to insert into the host lipid membrane [53]. Physical interaction studies have suggested that trehalose dimycolate (TDM) and glycopeptidolipide (GPL) insert into model membranes, such as liposomes, phospholipid monolayers and purified membranes of mitochondria [54]–[56]. This insertion may disturb the lipid composition of host membranes thereby altering their physical properties. In particular, these mycobacterial glycolipids have been shown to decrease the fluidity of membrane liposomes [54], as reported here for DIM. It remains unclear how DIM present in the envelope of *M.bovis* BCG or *Mtb* insert into the plasma membrane. Similarly, the relationship between DIM-induced changes in membrane organization and *Mtb* uptake are unknown. Changes in membrane lipid composition/organization are generally thought to affect a number of cell functions [40]. Only a few reports have described a clear association between the insertion of mycobacterial lipids and changes in plasma membrane functions. In particular, the insertion of TDM and GPL into membranes has been associated with the disruption of respiration and oxidative phosphorylation functions in isolated mitochondria [54],[56]. We showed that physical changes of the plasma membrane induced by cholesterol depleting drug drove bacteria toward a more-acidic compartment, as did DIM deficiency. Further studies are required to determine whether the effect of DIM on the control of phagosomal pH is a consequence of the initial reorganization of the plasma membrane described above. The fatty acid composition [57] and the incorporation of palmitic and oleic acid into macrophage membranes [58] have been shown to modify phagocytosis probably by inducing changes in conformation of certain proteins, including receptor [40]. We found that changes in the lipid organization of the plasma membrane induced by DIM altered the uptake of H37Rv, but not its binding. This suggests that the physical membrane effect of DIM preferentially affects the signalling pathway of phagocytic receptors rather than the recognition or binding of their ligands. These points remain unclear and are currently under investigation in our laboratory.

## Materials and Methods

### Antibodies, fluorescent probes, and reagents

Mouse monoclonal antibodies (Abs) raised against CD11b extracellular domain ICRF 44 and D12 (1∶10; IgG1 and IgG2a) and mouse anti-human Ab against the mannose receptor CD206 (1∶100; IgG1) were obtained from BD Biosciences (San Jose, USA). Mouse monoclonal Ab against human CD63 was obtained from Caltag Laboratories (Burlingame, USA). Rabbit polyclonal anti-serum against H^+^-ATPase proton pump was from Synaptic Systems (Göttingen, Germany). Irrelevant IgG1 and IgG2 were purchased from DakoCytomation (Glostrup, Denmark) and BD Biosciences, respectively. Anti-mycobacterium rabbit Ab was obtained as previously described [59]. Secondary Abs, LysoTracker Red DND-99 and Alexa Fluor 647-conjugated cholera toxin subunit B (Alexa-647 CTB) were purchased from Molecular Probes (Eugene, USA). 1,2-dipalmitoyl-sn-glycero-3-phosphocholine (DPPC) was purchased from Avanti Polar Lipids (Alabaster, USA). Py-met-chol was synthesized as previously described [42]. The purity of phospholipids and Py-met-chol was confirmed by thin-layer chromatography on silica gel (Merck, Darmstadt, Germany) using chloroform/methanol/water (65∶25∶4, v/v) as the solvent. For detections, we used Dittmer's reagent for DPPC [60] with heating to detect Py-met-chol. Lipids, dissolved in chloroform/methanol (9∶1, v/v) were stored at 4°C before use. The concentrations of phospholipid solutions were determined by a gravimetric approach using a Sartorius (Goettingen, Germany) super microbalance (10^−7^ g). Water was purified with a Milli-Q system (Millipore, Bedford, USA). All other chemicals were obtained from Sigma Chemical Co (USA).

### Bacterial strains and culture conditions

*M. tuberculosis* H37Rv (sequenced strain) and *M. bovis* BCG 1173P2 (Pasteur strain) were grown at 37°C on Middlebroock 7H9 broth (InVitrogen, Cergy Pontoise, France) supplemented with ADC (Becton Dickinson, Co, Sparks, USA). The strains were grown to mid-exponential growth phase and centrifuged at 1.000×*g* for 10 minutes at room temperature in a Jouan CR412 centrifuge with a T4 swing out rotor. Bacteria were then isolated as previously described [59]. Briefly, the medium was discarded, bacteria were dispersed by shaking for 1 minute with glass beads (4 mm diameter), and resuspended in PBS, pH 7.4. The remaining clumps were removed by allowing the bacterial suspensions to sediment for 10 minutes and centrifuging the supernatant for 10 minutes at 200×*g*. Bacteria were then counted under a microscope in a Thoma chamber and plated on 7H11 plates to establish more precise counts. When specified, bacilli were coated with purified DIM by suspending 5×10^7^ bacteria in 100 µl of 0.05% DIM in petroleum ether solution using a combination of published methods [20],[61]. Controls were prepared by treating bacilli with solvent alone or with 0.05% triglycerides in 100 µl of petroleum ether. The solvent was evaporated off and the bacteria were resuspended in PBS. Petroleum ether-treated H37Rv were as viable [62] and as capable to infect macrophages as the untreated strain (data not shown). Heat-killed H37Rv, used as a positive control for localization with acidic compartments in some experiments, were prepared by heating 5×10^7^ bacteria in PBS at 96°C for 1 h. Mycobacteria and zymosan were opsonised with human AB serum as previously described [59]. Suspensions of bacteria were then supplemented with 5% glycerol and stored at −80°C. For macrophage infection, bacteria were quickly thawed, vortexed and their titer adjusted to the required level in cell medium culture. Up to 85% of mycobacteria were free and identifiable, and their viability averaged 85%, as determined by the BacLight bacterial viability assay.

### Construction of recombinant *M. tuberculosis* H37Rv and *M. bovis* BCG strains

The *M. tuberculosis* H37Rv *ΔppsE::res-Ωkm-res* mutant (also named PMM56) was constructed in a previous study [48] by insertion/deletion within the *ppsE* gene which encodes a polyketide synthase required for the formation of the β-diol chain (Table 1). The corresponding *M. bovis* BCG *ΔppsE::res-Ωkm-res* (named PMM50) was constructed by the same method using the recombinant mycobacteriophage phWM01 [48].

For construction of the *M. bovis* BCG Δ*ppsB* mutant, we used the strategy described by Bardarov *et al.* [63]. Two DNA fragments (0.91 kb and 0.90 kb in length) overlapping the *ppsB* gene at its 5′ and 3′ extremities were amplified by PCR from *M. bovis* BCG genomic DNA by using primers ppsB1+ppsB2 and ppsB5+ppsB6, respectively (Table 1). The PCR was performed in a final volume of 50 µl containing 2.5 units of *Taq* DNA polymerase (New England Biolabs, Ipswich, MA), 10% Me_2_SO, and 1 µM of each primer. The amplification program consisted of heating for 10 minutes at 95°C followed by 30 cycles of 30 s at 95°C, 1 minute at 55°C and 3 minutes 30 s at 72°C with a final extension phase of 10 minutes at 72°C. These fragments were inserted into plasmid pGEM-T and a *res*-Ω*km-res* resistance cassette was inserted between these two PCR fragments to give pWM65. The PmeI fragment from pWM65, which contains the disrupted *ppsB::res*-Ω*km-res* gene was then inserted into the cosmid vector pYUB854 [63]. The resulting cosmid was cut with PacI and ligated with the mycobacteriophage phAE87. The ligation products were encapsidated *in vitro* using the Gigapack III XL kit (Stratagene) and the ligation mixture was used to infect *E. coli* HB101. Transfectants were selected on LB plates containing Km. A recombinant phagemid, named phWM07, harboring the disrupted gene construct was selected and transferred by electroporation into *M. smegmatis* for preparation of phage particles [63]. These particles were then used to infect *M. bovis* BCG and allelic exchange mutants were selected on 7H11 agar plates supplemented with OADC and Km. Several clones were analyzed by PCR using various primers (res1, res2, ppsB7, ppsB8, ppsB9, ppsB10) (Table 1) and one clone, named PMM92, giving an amplification pattern corresponding to allelic exchange was selected.

The *km* cassette was recovered by transferring the plasmid pWM19 containing the resolvase gene of transposon γδ into PMM92 [64]. The PMM92∶pWM19 transformation mixture was resuspended in 5 ml of 7H9 containing ADC and incubated for 48 h at 32°C. Transformants were selected directly in the liquid medium by adding hygromycin to the transformation mixture and incubating the culture at 32°C for 12 days. Viable bacteria were then recovered by plating serial dilutions on 7H11 plates without antibiotics and incubating at 39°C, a non permissive temperature for pWM19 replication. Several colonies picked randomly were then tested for growth on Km-containing plates. Colonies that were unable to grow on Km-containing plates but able to grow on antibiotic-free plates were selected and analyzed by PCR, using primers res1, res2, ppsB7, ppsB8, ppsB9 and ppsB10 (Table 1). One clone, PMM97, with an amplification pattern consistent with excision of the *km* cassette was retained for further analysis.

For complementation of the PMM100 mutant, we first recovered the antibiotic resistance cassette as decribed above. Four clones sensitive to kanamycin were analyzed by PCR using primers res1+ppsE4, res2+ppsE3, ppsE3+ppsE4 and conditions described in Simeone et al [48]. One clone displaying an amplification pattern consistent with excision of the *res-Ωkm-res* cassette was retained for further analysis and named PMM100 or H37Rv *ΔppsE::res*.

We constructed the plasmid pMVE as follows: the *ppsE* gene was amplified by PCR from *M. bovis* BCG genomic DNA, using oligonucleotides ppsE-ATG and ppsE-Stop (Table 1). The PCR product was purified, digested with NdeI and HindIII and inserted between the NdeI and *Hind*III sites of pMV361eHyg, a pMV361 derivative harboring the pBlaF* promoter from pMIP12 instead of the original *phsp60* promoter and carrying a *hyg* resistance marker [65],[66]. We used the plasmid pMVE for the electrotransformation of PMM100. Transformants were selected on plates containing kanamycin.

The lipids produced by the various recombinant strains were analyzed. We found that PMM56 and PMM100 did not synthesized DIM, whereas PMM100∶pMVE synthesized similar amounts of DIM to the wild type-strain, H37Rv (Figure S1). Similarly, the PMM50 and PMM97 strains synthesized no DIM and no PGL (data not shown).

The various *M. tuberculosis* and *M. bovis* BCG recombinant and wild-type strains were rendered fluorescent by the transfer of plasmid pMV361H *gfp* or pCG211. The plasmid pMV361H *gfp* was derived from the plasmid pMV361 [65] by the insertion of an hygromycin resistance cassette and the *gfp* gene. In this construct, *gfp* expression is under the control of the mycobacterial promoter *phsp60*. Plasmid pCG211 was constructed by insertion of the *gfp* gene between the BamHI and SpeI sites of plasmid pMIP12 [66]. The *gfp* gene was amplified from plasmid pMV361H *gfp* using primers gfp1 and gfp2.

### Cell culture

Human blood samples, obtained from the Etablissement Français du Sang of Toulouse (France), were collected from non tuberculous control donors. Peripheral blood monocytes were isolated as previously described [52] and cultured for 7 days on sterile glass coverslips in 24-well tissue culture plates (5×10^5^ cells/well) containing RPMI 1640 (Gibco, Cergy Pontoise, France) supplemented with 2 mM glutamine (Gibco) and 7% heat inactivated human AB serum. The culture medium was renewed on the third day. Macrophages derived from monocytes (MDMs) were washed twice with fresh RPMI medium before use.

The human promonocytic cell line THP-1 (ECACC 88081201; Salisbury, UK) was maintained in RPMI 1640 supplemented with 2 mM glutamine and 10% heat inactivated fetal bovine serum (FBS) at 37°C under an atmosphere containing 5% CO_2_. For differentiation into macrophages, cells were plated into 24 or 6-wells tissue culture plates (2×10^5^ and 10^6^ cells/well, respectively) containing a sterile glass coverslip and treated with 20 nM PMA for 48 h. Before use, cells were washed twice with fresh medium.

### Cell infection and phagocytosis assay

Infection of macrophages and phagocytosis were assessed, as previously described [52]. Briefly, macrophages were infected with GFP-expressing mycobacteria at the appropriate multiplicity of infection (MOI) in RPMI 1640. Infection was allowed to proceed at 4°C for binding or 37°C for uptake, and the extracellular bacteria were removed by 3 successive washes with fresh medium. Where specified, macrophages were initially treated with drugs for 30 minutes at 37°C, washed with fresh medium and infected with mycobacteria. The effects of drugs on cell viability and morphology were assessed by Trypan blue exclusion and light microscopy. At the end of the infection period, the cells were fixed for 1 h at room temperature with 3.7% paraformaldehyde in PBS supplemented with 15 mM sucrose, and aldehyde groups were neutralized with 50 mM NH_4_Cl. The cells were then rinsed in PBS, and extracellular mycobacteria were stained with rabbit anti-mycobacterium Ab (1∶50) which was detected by a Rhodamine Red-conjugated goat anti-rabbit secondary Ab. As the cells were not permeabilized, immunostained extracellular mycobacteria displayed red and green fluorescence and intracellular bacteria appeared as green fluorescent particles. Preparations were vizualized under a Leica DM-RB epifluorescence microscope to count and calculate (i) the number of bacteria per macrophages and (ii) the percentage of cells binding bacteria (% binding) or ingesting at least one particle (% internalization or phagocytosis). Where specified, data are also expressed as relative values, calculated as the percentage of internalisation or phagocytosis divided by control values. For each set of conditions, duplicate experiments were performed, and at least 100 cells per slide were counted.

### Colocalization experiments with Lysotracker, H^+^-ATPase, or CD63

Macrophages were infected with mycobacteria at an MOI of 10 for 1 h in RPMI 1640 at 37°C. Cells were then thoroughly washed and incubated with fresh RPMI 1640 supplemented with 7% human serum at 37°C under an atmosphere containing 5% CO_2_. We assessed LysoTracker Red colocalization, by washing macrophages at different time points after infection and incubating them with the acidotropic dye (1∶2000) in RPMI 1640 for 1 h. Cells were then rinsed, fixed with 3.7% paraformaldehyde for 1 h, washed and mounted on slides with DAKO mounting medium. For H^+^-ATPase or CD63 analysis, macrophages were fixed as described above, permeabilized by incubation with 0.3% Triton X-100 for 10 minutes at room temperature (RT), blocked by incubation with 0.3% BSA and incubated with rabbit polyclonal antiserum against H^+^-ATPase (1/100) or mouse anti-CD63 Ab (1∶100) for 1 h at RT, revealed with Rhodamine-Red conjugated goat anti-rabbit or anti-mouse Ab, washed and mounted on slides. Coverslips were viewed with a Leica DM-RB fluorescence microscope or a Leica TCS-SP2 confocal scanning microscope. All images were processed with Adobe Photoshop software. The fraction of phagosomes with GFP fluorescence colocalized with markers was determined by counting 100 phagosomes from at least 10 different fields in duplicate in at least three independent experiments for each time point.

### Staining of GM1 gangliosides

Macrophages were placed in contact with bacilli for 30 minutes at 4°C, washed with fresh medium and incubated with 50 µg/ml Alexa-647 conjugated CTB for 1 h at 4°C. Cells were then thoroughly washed, fixed by incubation in 3.7% paraformaldehyde, and visualized under a confocal scanning microscope.

### Labelling mixture preparation

MβCD was dissolved in water (683 mg in 7 ml) and heated to 70°C with shaking. A 1∶60 molar ratio (Py-met-chol/MβCD) was obtained by slowly adding 68 µl of a solution of Py-met-chol in dimethylformamide (138 mM). This solution was sterilized through filter with 0.22-mm pores and stored for period of up to several weeks at 4°C. The incubation medium consisted of 1 ml of this solution diluted (1∶30, v/v) in phosphate-buffered saline (PBS) without Ca^2+^/Mg^2+^. The final concentration of dimethylformamide was less than 0.05%. The final concentration of MβCD was 2.5 mM.

### Cell labelling

PMA-differentiated THP-1 plated in 6-well tissue culture plates (10^6^ cells/well) were washed twice with PBS without Ca^2+^/Mg^2+^ and incubated at room temperature for 5 minutes with 1 ml Py-met-chol/MβCD solution diluted 1/30. Cells were thoroughly rinsed with fresh PBS and then vigorously flushed with 1 ml PBS containing CaCl_2_ and MgCl_2_, resuspended at a final concentration of 5×10^5^ cells/ml and placed in contact with particles for 30 minutes at RT.

### Fluorescence spectroscopy

Emission spectra were recorded on a QuantaMaster-4 spectrofluorimeter (Photon Technology International, Birmingham, NJ) monitored with FeliX32 software. The excitation wavelength (bandwidth 5 nm) was set at 330 nm to avoid measurement of the Raman peak of water. The fluorescence emission (bandwidth 1 nm) was measured from 370 to 480 nm in 0.5-nm increments. The signal integration time was set to 200 ms. Fluorescence spectra were recorded in 1-cm quartz cuvettes in which the temperature was maintained at 45°C with a thermostat (Fisher Bioblock Scientific) for LUVs and at room temperature for THP-1 cell suspensions. *I*_1_, *I*_3_ and *I*_E_ were measured at 375, 386 and 470 nm, respectively.

### Preparation of lipid vesicles

Liposomes were prepared by mixing appropriate volumes of the stock solutions of DPPC, Py-met-chol and DIM (molar fractions were calculated according to the total quantity of lipids in a given suspension). Solvent was evaporated under a nitrogen stream and samples were desiccated for 2 h under vacuum. The dried lipid samples were hydrated in MOPS buffer (10 mM 3-morpholino-propanesulfonic acid, 100 mM NaCl, and 0.02% w/v NaN_3_, pH 7.4) and heated at 60°C for 20 min (temperature above than the gel-to-liquid transition temperature (Tm)). The sample was vortexed for 4 minutes to allow the formation of multilamellar vesicles (MLVs). MLVs (at final concentrations of 10^−4^ M) were sonicated for 10 minutes at a temperature above the Tm using a VibraCell sonicator (Sonics & Materials, Newtown, CT) with a titanium-plated tip delivering 12.5 W. The samples, constituted of small unilamellar vesicle suspensions, were immediately centrifuged for 25 minutes at 20,000×g (5408R centrifuge, Eppendorf, Hamburg, Germany) to remove any titanium particles. Small unilamellar vesicle suspensions were stored for 36 h to allow vesicle fusion. The sizes of relaxed large unilamellar vesicles (LUVs) were determined by measuring dynamic light scattering, using a Dynapro device (Wyatt Technology, Santa Barbara, CA). The LUVs in this study had a mean radius of 120 nm. However, the sample containing a 15.25 mol% of DIM presented a second peak in the DLS profile, corresponding to aggregates of unincorporated DIM. This molar percentage corresponds to the solubility limit of this lipid in a DPPC bilayer.

### Statistics

Data are presented as the mean±standard error of the mean (SEM) or standard deviation (SD) of the indicated number of experiments (n) performed in duplicate. The significance of differences was determined with the paired Student's t-tests.

## Supporting Information

Figure S1 Thin Layer chromatography analysis of DIM extracted from H37Rv, DIM-less mutants, and complemented strains. Lipids were labelled using [1-14C] propionate, extracted, and analyzed as described previously [48]. Three major bands were visible corresponding to three structural variants of DIM (dimycocerosates of phthiocerol, forms A and B, and dimycocerosates of phthiodiolone) [3]. These DIM were all missing in the PMM56, PMM100, PMM50, and PMM97 mutants. (A) TLC analysis of lipids extracted from the various *M. tuberculosis* WT and recombinant strains. (B) TLC analysis of lipids extracted from the various *M. bovis* BCG, WT and recombinant strains. (C) TLC analysis of DIM extracted from lived or heat-killed *M. tuberculosis*. Lipids were labelled using [1-14C] propionate, and half of the bacteria was heat-killed using the same conditions as the ones used for evaluation of acidification of *Mtb*-containing phagosome. Lipids were then extracted and analyzed as described previously [48].
(2.94 MB TIF)

Figure S2 DIM deficiency did not affect the ability of *M. tuberculosis* to replicate in human macrophages. MDMs were infected for 60 minutes with H37Rv WT (diamonds) or the PMM56 mutant (squares) at MOI 10, washed, and further incubated in the presence of serum. At various times after infection, MDMs were fixed and processed for differentiating intracellular and extracellular bacteria. Intracellular bacilli per macrophage were then quantified by counting 100 macrophages from at least 10 different fields. Values represented the mean+SEM of three independent experiments.
(0.11 MB TIF)
