# Supplementary figures and images for: Phthiocerol Dimycocerosates of M. tuberculosis Participate in Macrophage Invasion by Inducing Changes in the Organization of Plasma Membrane Lipids
Source: PLoS Pathog. 2009 Feb 6;5(2):e1000289. doi: 10.1371/journal.ppat.1000289 (PMC2632888; doi:10.1371/journal.ppat.1000289)

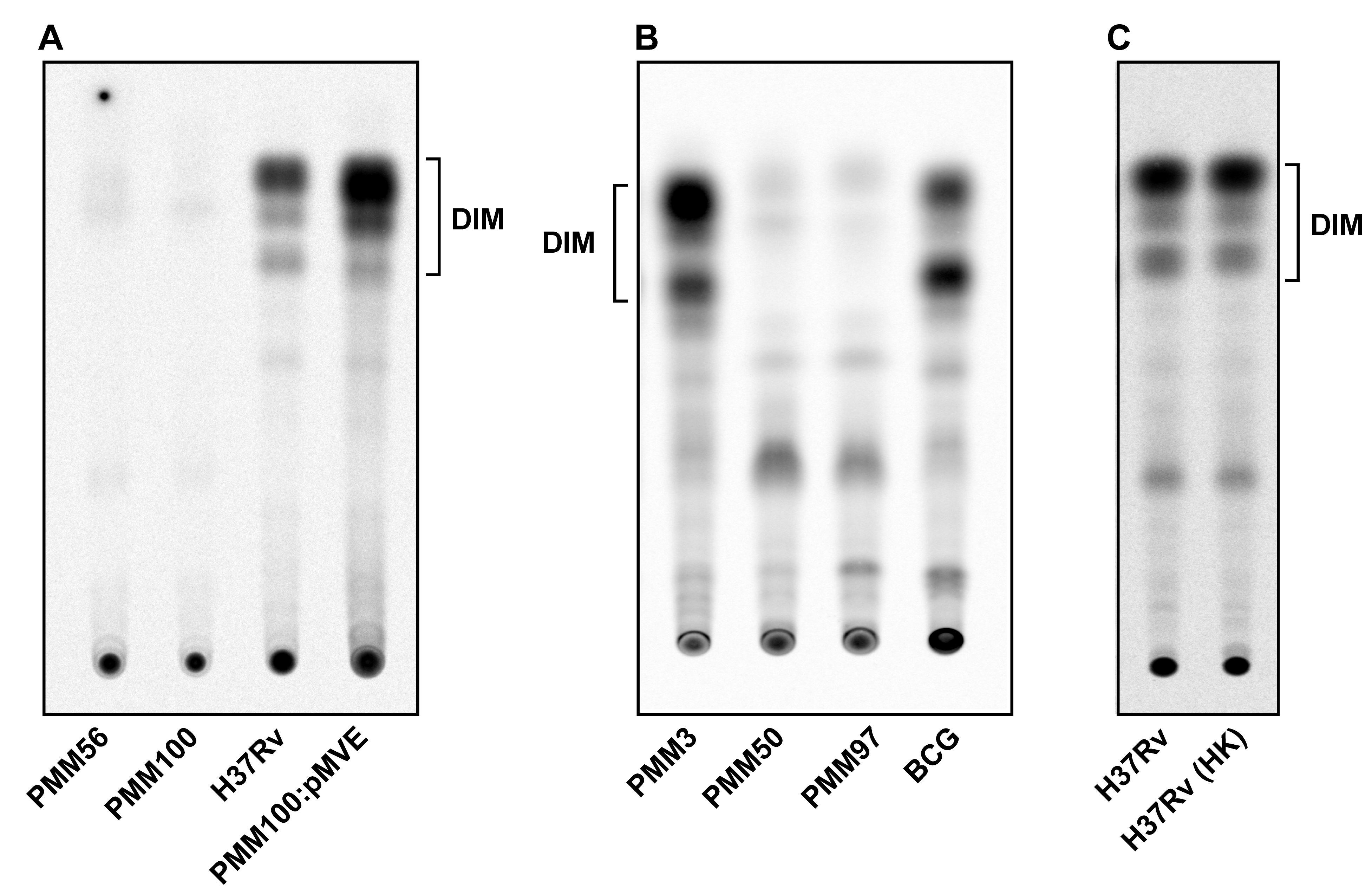

Supplement: Figure S1 — Thin Layer chromatography analysis of DIM extracted from H37Rv, DIM-less mutants, and complemented strains. Lipids were labelled using [1-14C] propionate, extracted, and analyzed as described previously [48]. Three major bands were visible corresponding to three structural variants of DIM (dimycocerosates of phthiocerol, forms A and B, and dimycocerosates of phthiodiolone) [3]. These DIM were all missing in the PMM56, PMM100, PMM50, and PMM97 mutants. (A) TLC analysis of lipids extracted from the various M. tuberculosis WT and recombinant strains. (B) TLC analysis of lipids extracted from the various M. bovis BCG, WT and recombinant strains. (C) TLC analysis of DIM extracted from lived or heat-killed M. tuberculosis. Lipids were labelled using [1-14C] propionate, and half of the bacteria was heat-killed using the same conditions as the ones used for evaluation of acidification of Mtb-containing phagosome. Lipids were then extracted and analyzed as described previously [48]. (2.94 MB TIF) [file ppat.1000289.s001.tif]

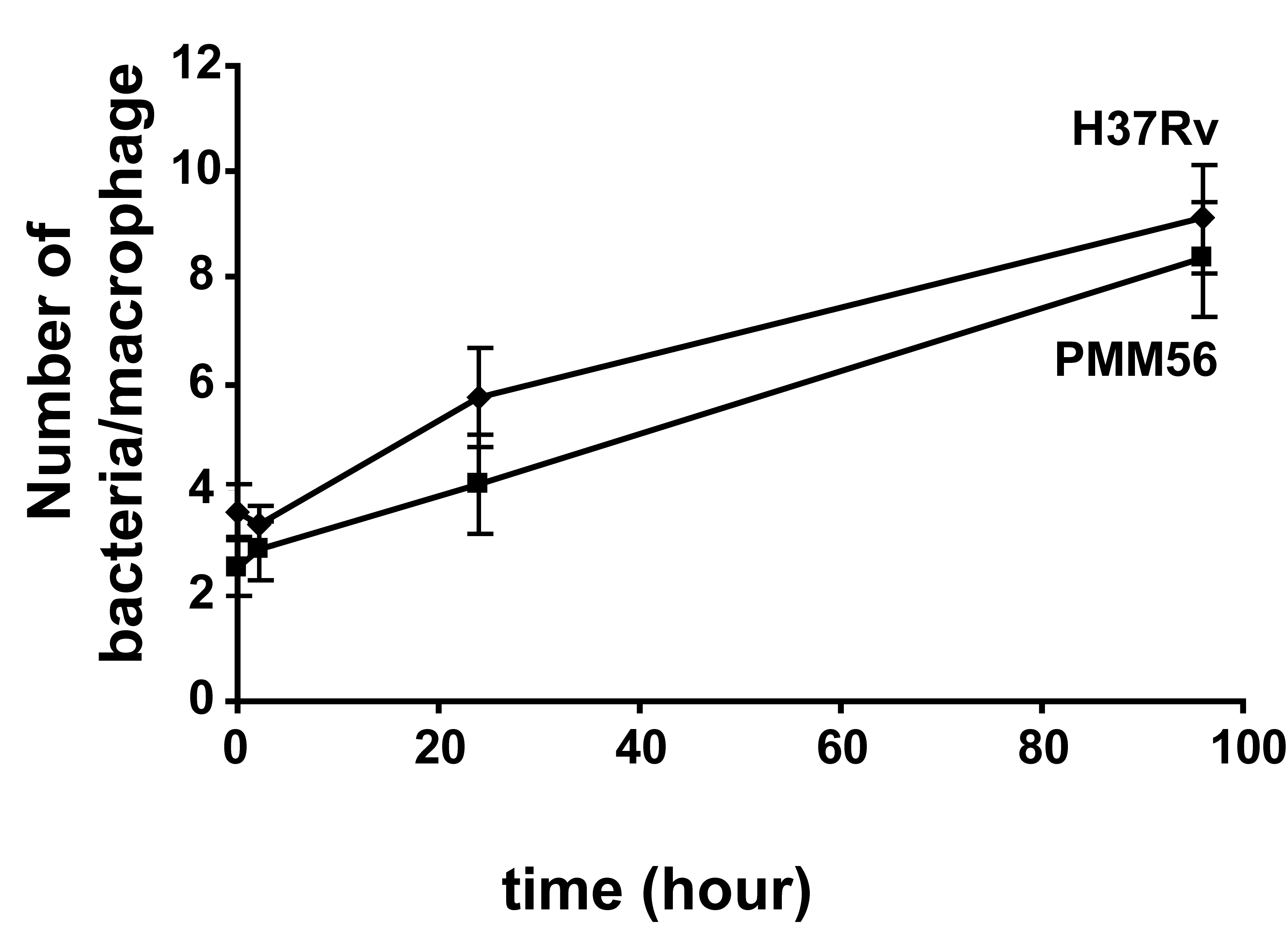

Supplement: Figure S2 — DIM deficiency did not affect the ability of M. tuberculosis to replicate in human macrophages. MDMs were infected for 60 minutes with H37Rv WT (diamonds) or the PMM56 mutant (squares) at MOI 10, washed, and further incubated in the presence of serum. At various times after infection, MDMs were fixed and processed for differentiating intracellular and extracellular bacteria. Intracellular bacilli per macrophage were then quantified by counting 100 macrophages from at least 10 different fields. Values represented the mean+SEM of three independent experiments. (0.11 MB TIF) [file ppat.1000289.s002.tif]
